# Need for Laboratory Ecosystems To Unravel the Structures and Functions of Soil Microbial Communities Mediated by Chemistry

**DOI:** 10.1128/mBio.01175-18

**Published:** 2018-07-17

**Authors:** Kateryna Zhalnina, Karsten Zengler, Dianne Newman, Trent R. Northen

**Affiliations:** a Lawrence Berkeley National Laboratory, Berkeley, California, USA; b University of California San Diego, San Diego, California, USA; c Center for Microbiome Innovation, University of California San Diego, San Diego, California, USA; d California Institute of Technology, Pasadena, California, USA; University of British Columbia

**Keywords:** chemistry of soil microbiomes, exometabolomics, laboratory ecosystems, metabolic networks, synthetic communities

## Abstract

The chemistry underpinning microbial interactions provides an integrative framework for linking the activities of individual microbes, microbial communities, plants, and their environments. Currently, we know very little about the functions of genes and metabolites within these communities because genome annotations and functions are derived from the minority of microbes that have been propagated in the laboratory. Yet the diversity, complexity, inaccessibility, and irreproducibility of native microbial consortia limit our ability to interpret chemical signaling and map metabolic networks. In this perspective, we contend that standardized laboratory ecosystems are needed to dissect the chemistry of soil microbiomes. We argue that dissemination and application of standardized laboratory ecosystems will be transformative for the field, much like how model organisms have played critical roles in advancing biochemistry and molecular and cellular biology. Community consensus on fabricated ecosystems (“EcoFABs”) along with protocols and data standards will integrate efforts and enable rapid improvements in our understanding of the biochemical ecology of microbial communities.

## Abstract (precis)

The chemistry underpinning microbial interactions provides an integrative framework for linking the activities of individual microbes, microbial communities, plants, and their environments. Currently, we know very little about the functions of genes and metabolites within these communities because genome annotations and functions are derived from the minority of microbes that have been propagated in the laboratory.

## PERSPECTIVE

**There are few studies more fascinating, and at the same time more neglected, than those of the teeming populations that exist in the dark realms of the soil. We know too little of the threads that bind the soil organisms to each other and to their world, and to the world above**.

Rachel Carson, *Silent Spring*, 1962

Microorganisms are on us and around us, catalyzing reactions on which we critically depend yet poorly understand. Their metabolism has been driving earth’s climate (1), building soils (2), and governing biogeochemical cycles for billions of years (3). The realization that each plant and animal in an ecosystem have evolved in the presence of microorganisms and is influenced by microbes greatly complicates understanding, predicting, and managing ecosystems. The health of many ecosystems is intimately connected to the health of soils, which are central to nutrient cycling in terrestrial ecosystems (4) and represent a vast reservoir of biodiversity (5).

Scientists have been studying soils for well over a hundred years. Each technical advance has seemingly revealed additional critical soil variables, including complex mineralogy, hydrology, pore architecture, chemistries, and biology, making soils one of the most heterogeneous biological systems on earth (6). Of the estimated 10^12^ microbial species on earth (7), soils are thought to support the greatest diversity of microbes, presumably as a result of the range of microenvironments found therein (8). Indeed, the spatial heterogeneity of soil facilitates a plethora of ecologic interactions that enable the evolution and maintenance of bacterial diversity (9, 10). How soil microbial communities living in diverse micron-scale niches interact with particles, various organisms, and plant roots to cycle roughly 50% of global organic carbon is still unclear (11). Simple models of homogeneous habitats cannot explain the speciation, dispersal, and biological interactions that exist in soil.

## CHEMISTRY IS THE LANGUAGE AND CURRENCY OF MICROBIAL COMMUNITIES

**FIG 1:**
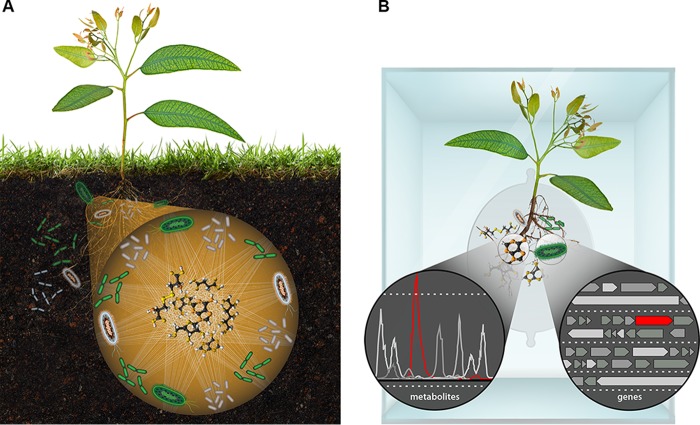
(A) Metabolites are the currency and communication for microbial communities. (B) Model laboratory ecosystems for discovering causal mechanisms of connections between genes and metabolites governing plant-microbe interactions.

Chemistry is what integrates the activities of individual microbes, microbial communities, plants, and other organisms and provides an important integrative framework for understanding soil microbiota (Fig. 1A). Individual cells consume and release small organic molecules (metabolites) and enzymes that modify their environment. These metabolites and enzymes are, in turn, modified by other organisms, and it is therefore the collective chemistry of microorganisms (12) that drives global processes (13).

Microbial metabolites range from simple molecules that serve as nutrients for other microbes or plants to complex molecules, like flavonoids, nonribosomal peptides, and polyketides that are associated with communication and antagonism of microbes within the environment (14). Metabolomic methods are now being directly applied to study soils and other complex environments (15). Unfortunately, most metabolites detected by mass spectrometry cannot be readily identified. This is because metabolite identification using tandem mass spectrometry, a leading approach, is based on comparison to reference compounds (16, 17). Hence, our understanding of earth’s chemical diversity is constrained to what we already know and have previously cataloged in various databases.

Yet we still have much to learn about the biological functions of known metabolites *in situ*. For example, even relatively simple metabolites such as phenazines, representatives of an extensive class of redox-active metabolites, can play very different roles depending on the microenvironment, from serving as antibiotics, to acting as signaling molecules, to facilitating energy generation, and mediating iron acquisition (18, 19). Whether an organism would be expected to resist, be harmed, or even benefit from phenazines depends not only on the state of the environment it inhabits (e.g., oxic or anoxic) but also on its metabolic state and biochemical ecology, i.e., whether it expresses specific phenazine-responsive genes that predict a particular fitness outcome and/or its proximity to other organisms that may degrade or chemically modify phenazines (20).

Each organism’s metabolic state thus represents a result of complex interactions between species and the environmental chemical landscape. Ultimately, the range of metabolites a microbe can produce and consume are encoded by its genome. Genomic analysis of the extremely small fraction (<1%) of soil microbes that have been cultivated in the laboratory (21) has revealed that a significant fraction of their genomes (up to 25%) is likely dedicated to the production of secondary metabolites (22), suggesting that these metabolites play a vital role for life in soil environments.

## METABOLIC “DARK MATTER”

Our understanding of microbial metabolism is largely based on a set of reductionist studies of model organisms, such as Escherichia coli, Bacillus subtilis, or Saccharomyces cerevisiae. Even for these well-studied microbes, more than 30% of their gene functions are currently unknown (23). Using DNA sequence homology, lessons from studies of these organisms have been extrapolated to all other microbes, including those observed in complex environments. Thus, essentially all metabolic processes we recognize are under the lamppost of characterized isolates that typically are grown quickly in the laboratory under nutrient-replete conditions. However, life in soil is restricted by many challenges, and thus, it would not be surprising if a vast yet uncharacterized metabolic “dark matter” associated with novel microbial metabolism and metabolic processes were to underpin the survival of soil microorganisms.

In the remainder of this perspective, we lay out a vision of integrated laboratory ecosystem experiments, experimental approaches, modeling, and analytic technologies that we believe have the potential to systematically advance our understanding of the biochemical ecology of microbial communities in complex environments. Included in this effort will be the integration of cutting edge omics techniques, including reconstruction of genomes from metagenomic sequences, which have proven valuable in advancing our understanding of the microbial potential in the environment (24). In addition, measurement of the metabolic activities and chemistry of metabolomes through metabolomic techniques will be critical. Yet omics approaches alone will not enable discovery of completely new functions and must be integrated with other approaches, such as classical physiology, genetics, and biochemistry.

## FABRICATING MODEL MICROBIAL ECOSYSTEMS IS NEEDED TO ADVANCE OUR UNDERSTANDING OF THEIR BIOCHEMICAL ECOLOGY

Sixty years ago, in the now classic review entitled “Biochemical ecology of soil microbes,” the eminent soil scientist M. Alexander suggested that the small size and relatively rapid generation time of microbes (versus higher organisms) naturally lends them to laboratory ecologic studies (25). However, while great advances have been made in environmental microbial genomics, our understanding of microbial community assembly, community structure-activity relationships, and responses to environmental perturbations is nascent. Studies of native microbial communities (e.g., in field studies) are limited by the high cost, complexity, and difficulty in controlling variables that present major challenges to deducing causal relationships, especially in ecosystems where sampling and measurements at temporal and spatial scales relevant for microbes are often not feasible. The most direct way to identify causal connections between members of the community and metabolites is to modulate a community and study its response. Yet, direct methods, such as deleting a prospective biosynthetic cluster of interest to determine its impact on other organisms, have been limited to laboratory environments.

Coupling advances in three-dimensional (3D) printing, sensors, and analytic and imaging technologies has the potential to enable ecosystem fabrication, producing “EcoFABs” that serve as a much-needed middle ground between model organisms and highly complex communities (26). The successful development of standardized, low-cost, and reproducible EcoFABs would greatly accelerate the discovery of the functional connections between microbes and metabolites. There are already a number of elegant studies on simple ecosystems that have provided important insights into the biochemical ecology of relatively simple environmental microbial communities (27–31). The further development of these approaches would provide a powerful complement to studies of native communities.

Native communities will be the inspiration and benchmark for EcoFABs, which in turn would enable perturbation, manipulation, and detailed observation not possible in native environments. Development of EcoFABs will depend on the formation of groups of investigators such as those that have coalesced around the study of single model organisms, such as the fly, mouse, worm, and zebrafish (32). However, not all microbial communities lend themselves to laboratory study due to the slow generation times of their members and/or the extreme conditions needed for them to grow. Adoption of reproducible standard model ecosystems that could be disseminated between scientists would greatly advance our understanding of biochemical ecology. Shared usage of model ecosystems would enable researchers to focus on their areas of interest and expertise, while permitting systematic comparisons with the results of others. Use of synthetic biology and genetic tools within these controlled laboratory environments would enable reductionist studies to determine gene and metabolite functions and discover new biochemistry. Principles and models developed could inform and be tested by field studies in an iterative fashion.

We believe that plants and their associated soil microbiota represent particularly attractive targets for ecosystem fabrication and synthetic community design. Such systems would enable investigation of diverse gene and metabolite functions relevant to microbe-microbe and plant-microbe interactions (Fig. 1B) (33). Initially, it would be desirable to build these EcoFABs using model plant species, such as *Arabidopsis* or *Brachypodium*, to leverage extensive knowledge of their biology, powerful genetic tools that have been developed, and advanced imaging technology. For example, the GLO-Roots technology enables visualization of the root system through thin soil sections (34).

Selecting community members for construction of standardized model ecosystems will require careful consideration to make the model ecosystems suitable for diverse lines of investigation. This will require achieving a balance of environmental relevance and experimental tractability (Table 1). Relevance should be based on observations of keystone species, known interactions, functional groups, and impact on important ecosystem properties in natural environments that are of broad interest and importance to the scientific community. To be tractable, microbes should be able to grow readily as isolates and be transformable. There is probably no group of isolates that will satisfy all of these criteria, so tradeoffs will be required and expedience is important. Significant effort has already been made to establish synthetic communities (28, 30, 35), and these findings and initial isolates may enable rapid progress. As the composition of these model communities is refined, it will be desirable to capture the dominant microbial taxa that have recently been correlated with specific environmental predictors across the globe (36). Yet we acknowledge that the question motivating any given study ultimately will dictate the organisms chosen as most relevant.

**TABLE 1:** Balancing environmental relevance with experimental tractability: examples of microbial features when selecting community members for construction of standardized model ecosystems

Selection criterion and microbial feature
Microorganisms should be relevant to natural ecosystems
Keystone species
Known biotic interactions
Impact on important ecosystem properties
Encompass major phyla and functional groups
Dominance across rhizosphere and soils
Microorganisms should be experimentally tractable
Grow in isolation
Genetically tractable
Existing resources and interest

Microfabrication technologies that permit control over the “microbial microenvironment” would provide a much-needed experimental platform for deconstructing microbial interactions. While this may sound futuristic, advances in 2D and 3D fabrication of biomaterials could rapidly enable the construction of microbial communities with carefully controlled microenvironments (37, 38), including those that capture aspects of real soil systems (39, 40). Critically, laboratory ecosystems can be constructed at a range of scales (e.g., aggregate scale, plant scale) and would permit application of existing genetic tools to test the roles of individual taxa and combinations of genes and microbes in more realistic laboratory conditions and associations, thus allowing causality to be probed.

## PROBING CAUSALITY WITHIN COMMUNITIES

Use of advanced technologies in the context of laboratory ecosystems will enable discovery of the relationships underpinning interactions within complex microbial networks. Critically, defined laboratory ecosystems will also help generate a common knowledge base, since all research is performed on a defined system with potentially modified parameters and conditions, allowing for expansion of compatible data and information. However, elucidation of who is communicating with whom requires innovative computational tools. Taking advantage of genomic and metabolomic information and data, genome-scale models have been deployed successfully to unravel complex and intertwined interaction in cocultures and simple communities (27, 41). These community systems biology approaches (42) benefit from decades of progress in the field of systems biology for single organisms (43). While community systems biology tools have been applied to predict interaction networks based on metabolite exchanges in cocultures and communities dominated by a few members (27, 44, 45), solving interaction networks of communities harboring tens or hundreds of members has been computationally challenging and will require the development of new algorithms and solvers.

**FIG 2:**
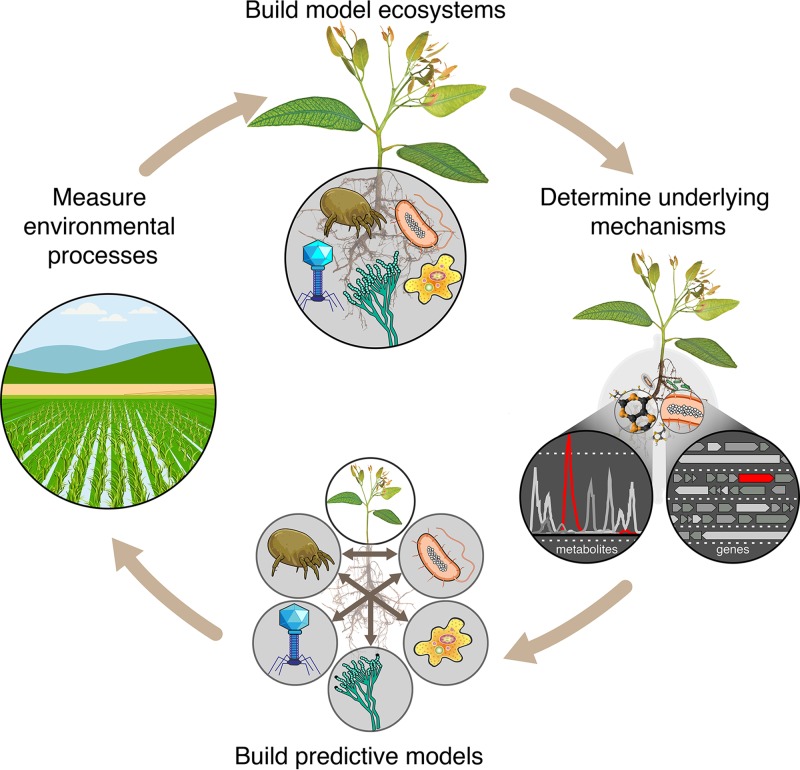
Dissecting environmental complexity with EcoFAB. Ecosystem fabrication (EcoFAB) workflow includes (i) analysis of environmental processes, (ii) building model ecosystems, (iii) identifying underlying mechanisms, (iv) developing predictive models, and (v) testing predictions in the field.

To rapidly advance our understanding of the biochemical ecology of microbiomes, we envision the workflow depicted in Fig. 2, where analysis of native ecosystems is used to design EcoFABs that in turn enable mechanistic studies and development of predictive models. The resulting predictions can then be tested in the field. Discrepancies identified between EcoFAB-derived predictions and field data can be used to iteratively refine the computational models. Starting small and systematically adding in new variables (both biological and abiotic) once the relationships between actors at the simplest scale are understood represents a rational approach to dissecting complex systems.

We see opportunities to integrate a range of innovative technologies within EcoFABs to discover and characterize the connections within communities and chemistry within soils. These techniques include, for example, sequence-based methods (46, 47), metabolomics (48), stable isotope probing (49–53) and metabolic labeling methods (54, 55), as well as trace gas analysis (56), advanced imaging techniques (57, 58), and DNA synthesis technologies (59). The latter is especially powerful in the context of EcoFABs, since low-cost DNA synthesis enables direct synthesis of genes and even whole biosynthetic pathways from metagenomes (60–64), allowing interrogation of these gene products in the lab. Whole pathways can now be cloned into host genomes and characterized using metabolomics to discover novel metabolites (63, 64) and enzyme activities (23, 61, 62, 65–68). Having defined consortia in these laboratory ecosystems would enable discovery of the ecologic effects of these metabolites. In addition, molecular engineering tools, such as transposon insertion sequencing (Tn-seq) and dual barcoded shotgun expression library sequencing (Dub-seq) (69, 70), and CRISPR-Cas9 tools (71, 72) could enable large-scale discovery of gene and metabolite functions within specific community and environmental contexts.

## CONCLUSION

The chemical signaling and metabolic webs of soil microbial communities are largely unexplored. To investigate the biochemical ecology of these environments, we need new technologies supporting reproducible ecosystem fabrication to enable use of observational and reductionist tools. Community consensus on a few EcoFABs along with protocols and data standards will enable discovery and detailed investigation of the “dark biochemistry” of microbial interactions. Specifically, it will support reproduction of the same microbial ecosystems in labs around the world, enabling researchers to build on each other’s results to advancing microbiome science much as model organisms have advanced our understanding of molecular and cellular biology. However, a critical challenge will be recapitulating sufficient complexity from native microbial communities to provide relevant insights, while at the same time making them experimentally tractable. Striking this delicate balance will require the engagement of scientists with diverse skills to design a series of model ecosystems designed with the appropriate level of complexity need to gaining a predictive understanding of how soil microbial ecosystems function.
